# Cannabinoid Receptors in Myocardial Injury: A Brother Born to Rival

**DOI:** 10.3390/ijms22136886

**Published:** 2021-06-26

**Authors:** Xinru Tang, Zheng Liu, Xiaoqing Li, Jing Wang, Liliang Li

**Affiliations:** Department of Forensic Medicine, School of Basic Medical Sciences, Fudan University, Shanghai 200032, China; xrtang18@fudan.edu.cn (X.T.); liuz18@fudan.edu.cn (Z.L.); lixq15@fudan.edu.cn (X.L.); wangj16@fudan.edu.cn (J.W.)

**Keywords:** cannabinoid receptor 1, cannabinoid receptor 2, myocardial injury, functional rival

## Abstract

Cannabinoid receptors typically include type 1 (CB1) and type 2 (CB2), and they have attracted extensive attention in the central nervous system (CNS) and immune system. Due to more in-depth studies in recent years, it has been found that the typical CB1 and CB2 receptors confer functional importance far beyond the CNS and immune system. In particular, many works have reported the critical involvement of the CB1 and CB2 receptors in myocardial injuries. Both pharmacological and genetic approaches have been used for studying CB1 and CB2 functions in these studies, revealing that the brother receptors have many basic differences and sometimes antagonistic functions in a variety of myocardial injuries, despite some sequence or location identity they share. Herein, we introduce the general differences of CB1 and CB2 cannabinoid receptors, and summarize the functional rivalries between the two brother receptors in the setting of myocardial injuries. We point out the importance of individual receptor-based modulation, instead of dual receptor modulators, when treating myocardial injuries.

## 1. Introduction

The endocannabinoid system (ECS) is a widely conserved lipid signaling system in mammals. Based on current knowledge, the ECS consists of cannabinoid receptors, endogenous cannabinoids (endocannabinoids), and the enzymes responsible for the synthesis and degradation of the endocannabinoids. Cannabinoid receptors typically include cannabinoid type 1 (CB1) and type 2 (CB2) receptors, and some recently identified orphan receptors such as GPR18, GPR55, and GPR119 [1] that exhibit limited sequence homology with CB1 and CB2 [2]. Six endocannabinoids have been recognized so far, namely anandamide (AEA), 2-arachidonoyl glycerol (2-AG), N-arachidonoyl-dopamine (NADA), 2-arachidonyl glyceryl ether (noladin ether), virodhamine (OAE), and lysophosphatidylinositol (LPI) [1,3], with AEA and 2-AG being the most active ones. In addition to endogenous ligands, exogenous cannabinoids such as natural phytocannabinoids and synthetic cannabinoids also bind to cannabinoid receptors [4]. There are currently over 100 different phytocannabinoids isolated from cannabis plants, with the most abundant one being delta-9 tetrahydrocannabinol (THC) which has a high affinity to both CB1 and CB2 [3]. The synthetic cannabinoids are a heterogeneous group of compounds that can be generally classified as dual CB1/CB2 receptor actions (non-selective), CB1-selective actions, and CB2-selective actions. WIN 55,212-2 and CP 55,940, for example, are common synthetic cannabinoids that show non-selectivity over CB1 and CB2 receptors. Arachidonyl-2′-chloroethylamide (ACEA), noladin ether, and arachidonylcyclopropylamide display higher affinity for CB1 than CB2, while AM 1241, JWH-133, and HU-308 display higher selectivity for CB2 than CB1 [4].

**Figure 1:**
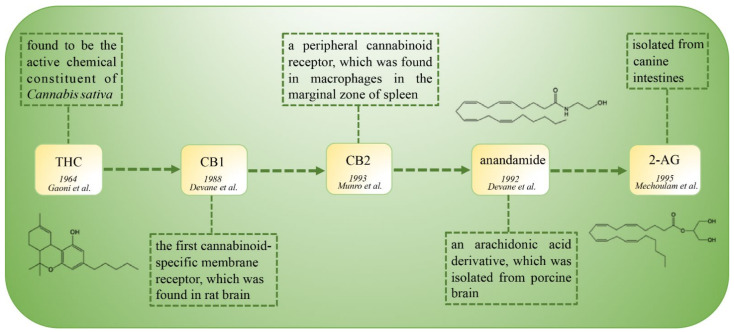
The timeline of the discovery of the cannabinoid family.

The discovery of cannabinoid receptors and their related ligands is an interesting journey (Figure 1). In the year 1964, THC was isolated and characterized as the active chemical constituent of *Cannabis sativa*. The target receptors of THC remained elusive for a long time until 1987, when the cDNA of CB1 (initially named SKR6) was cloned from a rat cerebral cortex cDNA library [5]. Interestingly, CB1 was initially considered as an orphan receptor due to a lack of ascertained ligands [6]. One year later (1988), CB1 was determined and characterized as the specific membrane receptor of a cannabinoid compound CP 55,940 [7], leading to the recognition of CB1 as the first cannabinoid receptor, instead of an orphan receptor [6]. Five years later (1993), a peripheral cannabinoid receptor was found in macrophages of spleen and verified to be also the target of exogenous cannabinoids. Hence, this peripheral cannabinoid receptor was adopted and named as CB2, holding a brotherhood with CB1 in the cannabinoid family. Due to the antecedent discovery and isolation, exogenous ligands were considered to be the “statutory guardians” (ligands) of these “orphans” (cannabinoid receptors). Then, at almost the same time that CB2 was identified, the endogenous ligands AEA and 2-AG were discovered in 1992 and 1995, respectively [8,9]. Due to endogenous ligand–receptor binding in physiological conditions, scientists then realized that the endogenous ligands should be the long-lost “immediate parents” (ligands) of these “orphans” (cannabinoid receptors). These seminal discoveries laid the foundations for research on the CB1 and CB2 cannabinoid receptors in both physiological and pathological conditions.

With more in-depth studies of the cannabinoid receptors (orphans “grew up”), scientists then realized that the two brothers looked to be largely different, not only in their appearance (sequence and structure), but also in their characters (molecular functions). This review generally introduces the basic differences between CB1 and CB2 cannabinoid receptors, and then summarizes the functional rivalry between the two brother receptors with emphasis on myocardial injuries.

## 2. General Differences of Cannabinoid Receptors

**Figure 2:**
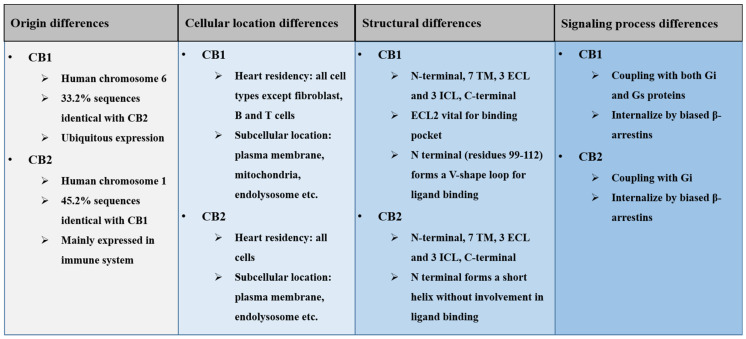
A summary of basic differences between the CB1 and CB2 receptors.

Generally, the brother receptors have many different and sometimes antagonistic characters in their origin, cellular distribution, structure, and downstream signaling pathways (Figure 2).

### 2.1. Origin Differences of Cannabinoid Receptors

**Figure 3:**
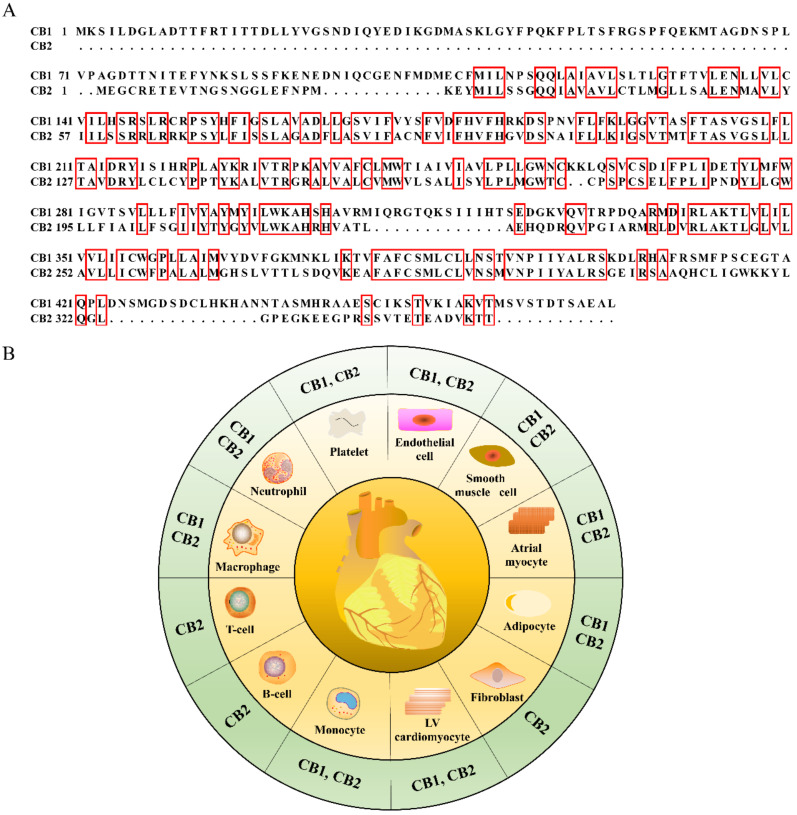
The amino acid sequence alignment of mouse CB1 with the CB2 receptor (**A**) and the cell tropism of CB1 and CB2 receptors within heart (**B**). The identical amino acid sequences are highlighted with red boxes in (**A**).

CB1 is encoded by the gene *CNR1* located on chromosome 6 (6q15, HGNC ID:2159) in *Homo sapiens*. CB2 is encoded by the gene *CNR2* located on chromosome 1 (1p36.11, HGNC ID: 2160) in *Homo sapiens* [4]. Only 33.2% of the CB1 sequences share similarity with the CB2 sequences, while only 45.2% of the mouse CB2 sequences share identity with CB1 (Figure 3A). After translation, CB1 is expressed throughout the body, highly in central nervous system (CNS), especially in the axons and presynaptic termini of neurons in the amygdala, hippocampus, cortex, basal ganglia outflow tracts, and cerebellum [10]. However, CB2 is mainly expressed in the immune system, astrocytes, and microglia in the CNS [11]. These origin differences have led to a conventional recognition of the CB1 as a central receptor and CB2 as a peripheral receptor [12], mirroring the difference in “birth” background of the brother receptors.

### 2.2. Cellular Location Differences of Cannabinoid Receptors

In addition to the organ distribution differences, cannabinoid receptors share great differences in cellular distribution within the heart (Figure 3B). Both CB1 and CB2 receptors share a similar localization in cell types such as cardiomyocytes, monocytes, adipocytes, atrial myocytes, smooth muscle cells, endothelial cells, platelets, neutrophils, and macrophages. However, the distribution spectrum of CB2 is wider than that of CB1, as CB2 further localizes in myocardial fibroblasts, B cells, and T cells. This difference underscores the potent involvement of CB2 in inflammatory responses by affecting immune cell attraction, macrophage polarization, and lymphocyte clusters in the pericardial adipose tissue [1].

CB1 and CB2 belong to the G-protein-coupled receptors (GPCRs) family, which have long been considered to localize on the cell surface. However, many researchers have suggested that GPCRs can also localize in the intracellular compartment and membranes [3]. It has been reported that CB1 can localize in the outer membrane of neuronal mitochondria and regulate neuronal energy metabolism [13]. Mutagenesis analysis identified the first 22 amino acids (amino acid residues 2–23) of the CB1 protein as responsible sequences for mitochondria localization [14]. CB1 can also localize in endosomal and lysosomal compartments [15,16]. CB2 has also been found to localize intracellularly in rodent medial prefrontal cortical pyramidal neurons [15] and specifically co-localizes with endolysosomes in U2OS cells [15].

### 2.3. Structural Differences of Cannabinoid Receptors

The first crystal structure of CB1 was reported in 2016 as a complex with antagonist AM6538 [17]. The overall architecture of the CB1 structure is comprised of seven transmembrane (TM) α helices (I to VII) which are connected by three extracellular loops (ECL1-3), an amphipathic helix VIII, and three intracellular loops (ICL1–3) [17]. The ECL2 region consists of 21 residues folding into an intricate structure projecting four residues, which are vital to mediating interactions with certain classes of ligands, and the two cysteines (Cys257 and Cys264) in ECL2 [18] into the binding pocket. The ELC3 region has a three-helical-turn extension of helix VII, thereby increasing the rigidity and probably decreasing the flexibility of the loop region in CB1 [17]. The overall CB2 structure also contains seven TM helices (I to VII), an amphipathic helix VIII, ECL, and ICL, generally similar to CB1 [19]. CB2 also exhibits a constrained conformation of ECL2, which is stabilized by a disulfide bond between two cysteines (Cys174–Cys179) [19]. The significant difference between CB1 and CB2 lies in helices I and II, which may influence the combination of antagonists. Besides, the non-truncated part of the N terminal in CB2 is in contrast to the V-shaped loop in CB1. The non-truncated part of the N terminal in CB2 forms a short helix over the orthosteric pocket with no direct involvement in antagonist binding [19], while part of the N terminal of CB1 (residues 99–112) forms a V-shaped loop that inserts into the ligand-binding pocket and plays a role in ligand binding [17].

The structural difference of both receptors is further supported by the observation that CB1 agonists share a high degree of conformational similarity with CB2 antagonists [19]. The synthetic CB2 antagonist AM10257, for example, has been verified to confer both CB1-selective agonism and high CB2-selective antagonism effects [19].

### 2.4. Signaling Difference of Cannabinoid Receptors

Both CB1 and CB2 can couple with Gi/o proteins to inhibit adenylate cyclase activity, causing decreases of intracellular cAMP levels [4] and leading to the dysregulation of downstream cascades (i.e., MAPK signaling [20,21]) controlled by protein kinase A (PKA) [22]. CB1 and CB2 receptors can also internalize through G-protein regulatory kinases/β-arrestins, and transduce signals to the Ras/MEK/ERK pathway through interaction with Gβγ and β-arrestin proteins [3]. However, unlike CB2, CB1 can also couple with Gs proteins to stimulate adenylyl cyclase activity, leading to receptor-mediated Ca^2+^ fluxes and phospholipase activations [21].

## 3. Functional Rivalries between Cannabinoid Receptors in Myocardial Injury

The mechanisms of the cardiovascular effects of CB1 and CB2 are complex, and may involve the modulation of autonomic outflow in the central and peripheral nervous systems as well as direct effects on the myocardium and vasculature [23]. Due to the above basic differences, the functions of the two brother receptors have many differences in myocardial injuries (Table 1). Generally, signaling through CB1 causes negative inotropy in heart, while CB2 causes positive inotropic effects [24,25].

### 3.1. Acute Myocardial Infarction (MI)

CB1 aggravates the inflammatory response in cardiac ischemic injury [20]. It has been reported that the CB1 antagonist rimonabant (also named as SR 141716A) is able to improve survival rate and restrict infarct size in rodents bearing left descending coronary artery ligation [23]. Additionally, one study showed that chronic daily rimonabant injection, initiated before and continued for 6 weeks after MI, could improve cardiac functions [20]. Currently, the mechanisms underlying CB1-antagonism-conferred protection against MI remain to be elucidated. Of note, as a representative CB1 antagonist, rimonabant has been marketed for antiobesity therapeutics and showed potent metabolism-modulation capacity [4]. Since systemic metabolic disorder is a high risk factor for myocardial ischemia and infarction, the mechanism of rimonabant-induced protection against MI may be a result of peripheral metabolism modulation rather than a direct effect on hearts [55].

In contrast, CB2 mitigates the inflammatory responses in cardiac ischemic injury [20]. The expression of CB2 increases in the situation of hypoxia and inflammatory stimulation [26]. Compared with wild-type (WT) mice, CB2^−/−^ mice had a more aggravated reduction in ejection fraction following MI [27]. In response to MI induced in WT mice, plasma and cardiac levels of the endocannabinoid 2-AG, but not AEA, palmitoylethanolamide, or oleoylethanolamide, were significantly elevated 24 h after infarction. The increased 2-AG promoted cardiac neutrophil and monocyte counts 24 h after infarction in WT mice but not in CB2^−/−^ mice [28], reinforcing the strong modulation of inflammatory responses by the 2-AG/CB2 axis. Modulation of the inflammatory responses by CB2 is mainly through directly affecting immune cell attraction, macrophage polarization, and lymphocyte clusters in the pericardial adipose tissue [20].

### 3.2. Cardiac Ischemia/Reperfusion (I/R) Injury

During early cardiac I/R injury, CB2 was upregulated in ischemic cardiomyocytes from WT mouse heart [29]. It has been reported that within hours of cardiac I/R, the activation of CB2 by its selective agonist JWH-133 exerted a potent anti-inflammatory effect, including the limitation of I/R infarct area and the increase of cardiac myocyte survival in response to stress [30]. The activation of CB2 is also the key to the reduction of leukocyte-dependent second-wave myocardial damage [31]. In a genetic depletion model, it has been reported that CB2^−/−^ mice have a more widespread injury than WT mice at 3 days [30] and 60 days following I/R injury [29]. In diabetic rats, CB2 activation can also attenuate I/R injury by counteracting tachycardia and restoring coronary perfusion pressure [32]. Besides, CB2 activation can promote lipopolysaccharide (LPS)-induced cardioprotective effects [33], and also enhance the protection effect of WIN 55,212-2 against cardiac I/R injury [32].

The cardioprotective effect of CB2 activation can be further evidenced by its antagonists. It has been reported that the CB2 antagonist SR 144,528 increases the infarct area in ischemic preconditioning models [33]. The CB2 antagonist SR 144,528 also blunts the protective effect of palmitoylethanolamide and 2-AG on hearts in rat cardiac I/R injury models [34], whereas the CB2 agonist JWH-015 enhances the cardiac protection of palmitoylethanolamide by activating the p38/ERK 1/2 kinases and PKC signaling [34]. Further, although the treatment of animals with the CB1/CB2 dual agonist WIN 55,212-2 30 min before induction of cardiac I/R significantly reduced the extent of infarct size in the area at risk, the selective CB2 antagonist AM 630 but not the selective CB1 antagonist AM 251 abolished the protective effects of WIN 55,212-2 [31], strongly enhancing the notion that CB2 dominantly protects against the I/R injury. In fact, the CB2 antagonist AM 630 alone produced a slight but significant increase in infarct size compared with vehicle alone [31], suggesting the endogenous cardioprotection of CB2 under physiological states. Of note, although the pharmacology of 2-AG and AEA is quite similar, in that both can bind to and stimulate CB1 and CB2 receptors [56], it is 2-AG and not AEA that has received wide attention for its protective effects on I/R injury, predominantly via a CB2-dependent manner [35,56]. The endocannabinoid AEA has only been recently found to transiently increase in mouse heart undergoing early I/R injury [29], and treatment of the AEA (1 mM) significantly reduced infarction of the left ventricle by 10% in rat isolated hearts subjected to I/R injury. However, the infarct-limiting action of AEA was not mimicked by agonists selective for CB1 or CB2 receptors, suggesting the involvement of a novel cannabinoid mechanism beyond the CB1 and CB2 receptors [35].

Unlike the well-recognized cardioprotection of CB2, the biological function of CB1 remains controversial in cardiac I/R injury. In some research, the CB1 antagonist rimonabant has been demonstrated to protect against cardiac I/R injury [36]. On the other hand, another CB1 antagonist, AM 251, has also been proven to further aggravate cardiac I/R injury [37]. This controversial finding may be explained by the fact that CB1 will be desensitized after chronic endocannabinoid elevations. The upregulation of CB1 in hearts only occurs in the early stage of MI, while the upregulation of CB2 can be monitored in both acute and late phases of MI [20]. Thus, it is much harder to study CB1 than CB2.

### 3.3. Pathological Cardiac Hypertrophy

**Figure 4:**
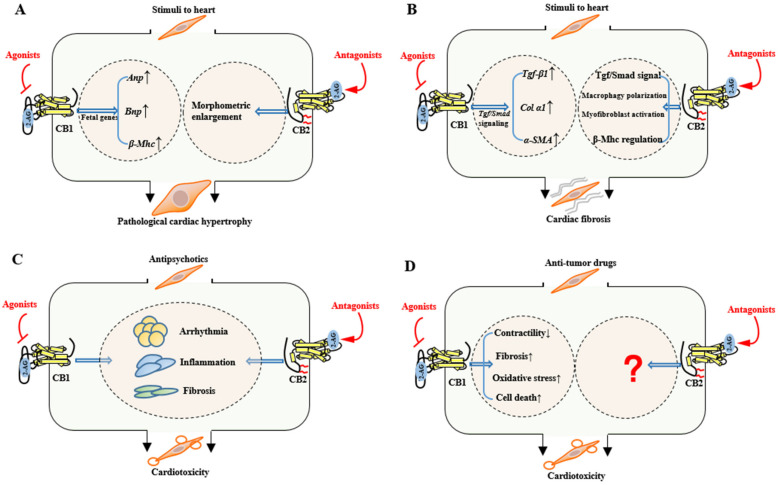
Summary of the functional differences between cannabinoid CB1 and CB2 receptors in pathological cardiac hypertrophy (**A**), cardiac fibrosis (**B**), antipsychotic cardiotoxicity (**C**), and anti-tumor drug cardiotoxicity (**D**). The 2-AG is depicted as a representative endogenous ligand of cannabinoid receptors. ↓ indicates inhibition and ↑ indicates promotion.

Inflammatory response is a significant pathological process to cardiac remodeling [57]. It has been reported that predominant expression of CB2 on cardiomyocytes associates with persistent inflammation and active remodeling in hypertrophic myocardium of patients with aortic stenosis [57]. Interestingly, both CB1 and CB2 mediate R-methanandamide-suppressed hypertrophic indicators. However, the selective CB2 agonist JWH-133 prevented only myocyte enlargement but not brain natriuretic peptide gene activation, while the CB1/CB2 dual agonist CB-13 inhibited both hypertrophic indicators [38], indicating that the two brother receptors individually suppress myocyte enlargement and fetal gene activation, respectively (Figure 4A). Furthermore, CB2^−/−^ mice showed vulnerability to pro-inflammatory responses such as higher macrophage infiltration and lower IL-10 expression than WT mice after left pulmonary artery occlusion, and accordingly showed stronger cardiomyocyte hypertrophy, presenting with higher tenascin-C expression and lower reactive oxygen scavenger enzymes induction than WT hearts [39].

Similar to the CB2 agonists, the CB1 antagonist rimonabant (or SR 141716A) attenuates left ventricular hypertrophy in chronic kidney disease mice by the upregulation of Akt phosphorylation [40]. The selective CB1 antagonist rimonabant also prevents adverse cardiac remodeling and improves cardiac functions after ischemic injury [23].

Of note, though the majority of the literature agrees that CB1 antagonists confer an anti-hypertrophic property, the CB1/CB2 dual agonist CB-13 inhibited both myocyte enlargement and fetal gene expression [38], indicating that CB1 protects against cardiac hypertrophy, which is in contrast to the majority of reports. We hypothesize that this inconsistent result may suggest the protective effect of CB1 on pathological hypertrophy being related to CB2, since in the condition of sole activation of CB1 and the absence of CB2 signaling, myocyte hypertrophy will persist and potentially give rise to adverse endpoints such as ischemia [38]. Additional evidence supporting the dominant position of CB2 in pathological cardiac hypertrophy lies in the fact that only CB2 exclusively locates in myocardial fibroblast, B cells, and T cells (Figure 2). The cell-type-specific expression of CB2 provides a biological basis for the direct modulation of inflammatory responses and the following adverse cardiac remodeling.

### 3.4. Cardiac Fibrosis

Cardiac fibrosis is an unwelcome consequence of multiple stimuli to myocardium, and seriously curbs cardiac diastolic and systolic functions, ultimately leading to heart failure [58,59]. TGF-β1/Smad signaling is a main pro-fibrotic pathway that drives fibrogenesis in heart [41,60]. In addition, crosstalk between the TGF-β/Smad signaling and other non-canonical pathways such as Wnt/β-catenin [61], EGFR signaling [62,63], and mTOR [64,65] regulates myocardial fibrogenesis in a synergistic way.

Multiple sources of pharmacology-based evidence have suggested the critical involvement of CB1 in the cardiac fibrogenesis process [4]. As a G-protein-coupled receptor, CB1 activates signal transduction and mediates fibrogenesis mainly through the TGF-β1/Smad3 pathway (Figure 4B). CB1 manipulates the transcription of pro-fibrotic molecules such as collagens, fibronectin, and α-smooth muscle actin (α-SMA) [41,42], and selective CB1 neutral antagonists AM 6545 and AM 4113 interfere with TGF-β1-mediated inflammation and fibrosis [42]. In an experimental model of chronic kidney disease, the expression of pro-fibrotic factors such as collagen α1, TGF-β1 precursor, and α-SMA were evidently reduced in myocardium after the pharmacological blockade of CB1 [40]. In a diabetic model, selective CB1 antagonists rimonabant (or SR 141716A) and AM 281 prominently ameliorated myocardial fibrosis, as evidenced by decreased collagen deposition and the downregulation of mRNA markers of fibrotic factors such as collagen-1, fibronectin, and TGF-β1 [27]. Blockade of CB1 signaling by rimonabant decreased MMP-9 activity and TGF-β1 expression in rat myocardium with experimental metabolic syndrome, and extenuated the extracellular matrix deposition and fibrosis progression [23]. These small molecules are CB1-selective antagonists that should not confer off-target effects. Therefore, the above pharmacology-based studies conclude that CB1 is a mediator of myocardial fibrosis.

In great contrast to CB1, CB2 displays a potent protection against fibrogenesis and delays cardiac remodeling processes (Figure 4B). The activation of CB2 signaling by AM 1241 retarded myocardial fibrosis during the post-myocardial infarction phase via accelerating the translocation of the fibrogenesis-associated transcription factor Nrf2 to nucleus and blocking the TGF-β1/Smad3 pathway [43]. In fact, CB2 is found to be indispensable for complete functional recovery and morphological regression of fibrosis in cardiac repair from I/R injury. After genetic knockout of CB2 (CB2^−/−^), reversible collagen III was lowered and irreversible collagen Iα was more prominent as compared to WT mice [29]. Moreover, myocardial fibrosis was intensified following four-week I/R injury after genetic depletion of cardiac CB2, as featured by positive TGF-β1 staining and expanded fibrotic scars [30]. All these pharmacological and genetic approaches suggest the endogenous cardioprotection of CB2 towards myocardial fibrosis.

Mechanistically, unlike CB1, which mediates myocardial fibrosis via regulating cardiac TGF-β/Smad signaling, CB2 blunts myocardial fibrogenesis in a more sophisticate manner (Figure 4B). CB2 could exert its TGF-β1-dependent antifibrogenic property [30,44]. It further regulates the expression of β-isoform myosin heavy chain to decrease contractile velocity under pressure overload in hearts [45]. CB2 also mitigates myocardial injury via manipulating macrophage polarization to maintain M1/M2 macrophage balance [46]. Due to the additional localization in fibroblast, CB2 also directly regulates the myofibroblast activation and thereby defending unwelcome myocardial fibrosis [30].

### 3.5. Miscellaneous Myocardial Injury

#### 3.5.1. Antipsychotic Cardiotoxicity

Second-generation antipsychotics (SGAs) including clozapine, olanzapine, and quetiapine are potent drugs for treating mental disorders such as schizophrenia, bipolar disorder, and major depressive disorders [66]. Nevertheless, antipsychotic-induced cardiotoxicity has been frequently observed and gradually cited as a major concern in long-term clinical practice [67] and in forensic autopsy [68,69]. According to a population-based study, current users of SGAs had higher rates of sudden cardiac death than non-users, with an adjusted incidence-rate ratio of 2.26 [70].

While the knowledge regarding antipsychotic cardiotoxicity derives largely from clinical and autopsy observations, deep mining of the molecular mechanisms has a long way to go. We have shown that dysregulated spliceosome signaling paved common ways for representative SGA cardiotoxicity, and the pharmacological blockade of a GPCR histamine 1 receptor (HRH1) only partially rescued the spliceosome signaling [47], leaving a wide space for the conception of other GPCRs’ involvement in antipsychotic cardiotoxicity [48]. This has led us to identify the cannabinoid receptors as critical modulators of antipsychotic cardiotoxicity in independent mouse models (Figure 4C). Upon chronic clozapine or quetiapine stimuli, serum levels of both AEA and 2-AG significantly decreased in mice, and CB1 translocated to cytoplasm, whereas CB2 remained in the plasma membrane of myocytes [49,50]. In the clozapine-insulted mouse model, CB1 antagonists (rimonabant and AM 281), but not its agonist ACEA, significantly attenuated clozapine-induced cardiac dysfunction; the same was seen for selective CB2 agonists (JWH-133 and AM 1241) but not its antagonist (AM 630). The extent of clozapine-induced cardiac fibrosis and serum levels of inflammatory cytokines were accordingly decreased by these beneficial compounds [49]. Similarly, in the quetiapine-treated mouse model, pretreatments with CB2R agonists JWH-133 and AM 1241 or CB1R antagonists rimonabant and AM 281 led to a relief in quetiapine-induced myocardium toxicity, particularly the quetiapine-induced myocyte necroptosis [50]. These pharmacological studies suggest the critical yet opposite functions of cannabinoid receptors in antipsychotic cardiotoxicity.

Of particular interest, antipsychotic use also raises critical concern regarding patients’ weight gain and metabolic disorders. There is also a strong link between CB1 and energy intake/storage, as well as glucose/lipid metabolism [71]. Hence, CB1 antagonists seem to confer dual actions, one to be marketed for weight loss and the second to be tested for improving cardiovascular outcomes in patients with long-term antipsychotic use [72]. However, rimonabant—a representative CB1 antagonist that was withdrawn after being marketed—was also reported to cause serious psychiatric disorders [72]. It is therefore mandated to carefully select low-toxicity yet sufficiently effective CB1 antagonists for the clinical intervention of antipsychotic cardiotoxicity.

#### 3.5.2. Anti-Tumor Drug Cardiotoxicity

The toxic side effects of most chemotherapeutic agents have been widely acknowledged in clinical practice, stimulating the rapid update of anti-tumor drugs [73]. However, anthracyclines such as doxorubicin (DOX) are still important in first-line treatment for breast cancer, lymphoma, sarcoma, and childhood hematological malignancy, owing to a lack of effective alternatives [74]. The subclinical damage caused by the cardiotoxic effect of anthracyclines, especially doxorubicin (DOX), is most frequently observed [75,76,77].

CB1 is a crucial mediator of DOX-induced cardiotoxicity (Figure 4D). In CB1 knockout mice, DOX-induced increases in left ventricular end-diastolic pressure, prolongation of relaxation time constants, and cardiac fibrosis were markedly alleviated [51]. CB1 knockout mice also presented resistance to DOX-induced left ventricular dysfunction, oxidative/nitrosative stress, antioxidant defense impairment, MAPK signaling activation, as well as cell death and/or fibrosis in hearts [51]. Likewise, CB1 antagonists exhibited beneficial effects on improving the DOX-induced depression of load-independent indexes of cardiac contractility such as PRSW (preload-recruitable stroke work) and ESPVR (end-systolic pressure–volume relation) [52]. In turn, when the endocannabinoid AEA or the synthetic CB1 agonist HU-210 was co-administered with DOX, the DOX-induced MAPK activation and cell death were significantly enhanced [51]. At the cellular level, the CB1 antagonists’ beneficial effects were accompanied with blockade of early apoptosis in rat H9C2 cells [52]. Interestingly, CB2 does not seem to be involved in DOX cardiotoxicity, according to the currently available literature. This raises further questions as to whether CB1 and CB2 have additional unidentified distinction (i.e., affinity differences) as to anti-tumor drugs.

#### 3.5.3. Ethanol-Induced Myocardial Injury

Chronic ethanol exposure can impair the myocardium, leading to irreversible cardiomyopathy as evidenced by progressive reduction in myocardial contractility, high level of creatine kinase, and interstitial fibrosis [53]. It has been reported that 30-day ethanol exposure significantly increased the serum level of AEA by 3–4-fold, while the serum level of 2-AG showed a slight increase at this time and then tended to decrease after longer exposure [54]. Pharmacological activation of CB2 protected against ethanol-induced myocardial injury and myocyte necroptosis, while CB1 seemed to be less involved when compared with CB2-mediated effects. Selective CB2 agonists JWH-133 and AM 1241 notably improved cardiac function in 45-day continuous alcohol exposure. The mRNA expression of pro-fibrotic factors and the necroptotic phosphorylating cascade were also significantly alleviated by the CB2 agonists but not its antagonist AM 630 [54]. The CB2-dominant involvement in ethanol toxicity again raises the question as to whether CB1 and CB2 have additional differences beyond those depicted in Figure 2.

## 4. Conclusions

This review introduced the general differences of the two brother receptors CB1 and CB2, and summarized their functional differences and rivalries in multiple myocardial injuries. Current pharmacological and genetic approaches have documented CB1 as an injury mediator, while CB2 is born to combat CB1’s detrimental effects and serves as an endogenous cardioprotective receptor in most myocardial injuries, with the exception of DOX and ethanol-induced cardiotoxicity where CB1 and CB2 individually dominate the cardiotoxic effects. Due to the functional rivalry, this review also points to the notion that the dual agonism or antagonism of cannabinoid receptors may not be necessarily clinically efficacious, and the treatment of myocardial injuries might only be beneficial when based on single-receptor activation or inhibition.

## Figures and Tables

**Table 1:** A summary of the functional rivalry between CB1 and CB2 receptors in myocardial injuries.

Category	CB1 Function	CB2 Function	References
Myocardial infarction	CB1 aggravated cardiac ischemic injuries	CB2 mitigated cardiac ischemic injuries	[20,23,26,27,28]
Cardiac I/R injury	Majority of the literature documents CB1 as a mediator of I/R injury, although there is some controversy across studies	CB2 potently protected from I/R injury	[29,30,31,32,33,34,35,36,37]
Pathological cardiac hypertrophy	Majority of the literature documents CB1 as a pro-hypertrophic receptor, and CB1 tended to be not as potent as CB2 in controlling hypertrophy	CB2 potently conferred anti-hypertrophic property	[38,39,40]
Cardiac fibrosis	CB1 promoted fibrogenesis mainly through TGF-β1/Smad3 pathway	CB2 ameliorated cardiac fibrosis via TGFβ1-dependent and independent manners	[23,27,29,30,40,41,42,43,44,45,46]
Antipsychotics cardiotoxicity	Pharmacological inhibition of CB1 was cardioprotective	Pharmacological activation of CB2 was cardioprotective	[47,48,49,50]
Anti-tumor drug cardiotoxicity	Genetic ablation or pharmacological antagonism of CB1 was cardioprotective	Unknown	[51,52]
Ethanol-induced myocardial injury	Less known	CB2 attenuated ethanol toxicity	[53,54]

## Data Availability

Not applicable.

## Abbreviations

CB1	cannabinoid receptor type 1
CB2	cannabinoid receptor type 2
CNS	central nervous system
ECS	endocannabinoid system
AEA	anandamide
2-AG	2-arachidonoyl glycerol
NADA	N-arachidonoyl-dopamine
OAE	virodhamine
LPI	lysophosphatidylinositol
THC	delta-9 tetrahydrocannabinol
GPCRs	G-protein-coupled receptors
ECL	extracellular loop
ICL	intracellular loop
PKA	protein kinase A
WT	wild type
MI	myocardial infarction
I/R	ischemic/reperfusion
LPS	lipopolysaccharide
α-SMA	α-smooth muscle actin
SGAs	second-generation antipsychotics
DOX	doxorubicin
PRSW	preload-recruitable stroke work
ESPVR	end-systolic pressure–volume relation
TM	transmembrane
